# Oxidation Behavior of Refractory AlNbTiVZr_0.25_ High-Entropy Alloy

**DOI:** 10.3390/ma11122526

**Published:** 2018-12-12

**Authors:** Nikita Yurchenko, Evgeniya Panina, Sergey Zherebtsov, Gennady Salishchev, Nikita Stepanov

**Affiliations:** Laboratory of Bulk Nanostructured Materials, Belgorod State National Research University, Belgorod 308015, Russia; yurchenko_nikita@bsu.edu.ru (N.Y.); 985376@bsu.edu.ru (E.P.); zherebtsov@bsu.edu.ru (S.Z.); salishchev@bsu.edu.ru (G.S.)

**Keywords:** high entropy alloys, refractory, oxidation, complex oxides

## Abstract

Oxidation behavior of a refractory AlNbTiVZr_0.25_ high-entropy alloy at 600–900 °C was investigated. At 600–700 °C, two-stage oxidation kinetics was found: Nearly parabolic oxidation (n = 0.46–0.48) at the first stage, transitioned to breakaway oxidation (n = 0.75–0.72) at the second stage. At 800 °C, the oxidation kinetics was nearly linear (n = 0.92) throughout the entire duration of testing. At 900 °C, the specimen disintegrated after 50 h of testing. The specific mass gains were estimated to be 7.2, 38.1, and 107.5, and 225.5 mg/cm^2^ at 600, 700, and 800 °C for 100 h, and 900 °C for 50 h, respectively. Phase compositions and morphology of the oxide scales were analyzed using X-ray diffraction (XRD) and scanning electron microscopy (SEM). It was shown that the surface layer at 600 °C consisted of the V_2_O_5_, VO_2_, TiO_2_, Nb_2_O_5_, and TiNb_2_O_7_ oxides. Meanwhile, the scale at 900 °C comprised of complex TiNb_2_O_7_, AlNbO_4_, and Nb_2_Zr_6_O_17_ oxides. The oxidation mechanisms operating at different temperatures were discussed and a comparison of oxidation characteristics with the other alloys was conducted.

## 1. Introduction

The so-called high-entropy alloys based on the refractory elements (refractory high-entropy alloys, RHEAs) are considered as candidates for high-temperature applications [1,2,3,4,5]. Some of them have high strength at T ≥ 1000 °C and thus can potentially operate at higher temperatures than commercial nickel-based superalloys [2,5,6,7,8,9,10,11,12,13]. Some RHEAs are sufficiently ductile at room temperature even in tension [14,15,16,17,18] and can be cold rolled [19,20,21,22]. However, refractory elements and their alloys are generally vulnerable to oxidation. A number of studies focused on oxidation behavior of different RHEAs [23,24,25,26,27,28,29,30,31,32,33] have demonstrated that some of them possess much better oxidation resistance than conventional refractory alloys; however, the oxidation behavior strongly depended on the alloy composition and testing conditions. Note that the vast majority of the performed research was conducted at temperatures of T ≥ 1000 °C [23,24,25,26,27,28,33].

Much less attention has been paid to the oxidation resistance of RHEAs at lower temperatures. For instance, an oxidation behavior of a series of Al_x_TiZrNbHfTa (x = 0–1) RHEAs was examined at temperatures of 700, 900, 1100, and 1300 °C [22]. It was found that at 700 and 900 °C the TiZrNbHfTa alloy exhibited rapid oxidation—the so-called pesting phenomenon. The oxide layer detached easily from the bulk material and the formation of voids on the specimen surface was observed. An addition of Al increased the oxidation resistance and suppressed pesting. Similar behavior was found in the Hf_0.5_Nb_0.5_Ta_0.5_Ti_1.5_Zr alloy at 600–1000 °C [20]. Bulk samples of the alloy disintegrated into powder already after 5 h of oxidation. Therefore, additional studies of the oxidation behavior of RHEAs at T < 1000 °C are required.

These studies are especially needed for RHEAs, which have a potential for operation at temperatures below 1000 °C. For instance, one of the perspective alloys of this group of RHEAs can be the AlNbTiVZr_x_ alloys and, particularly, the AlNbTiVZr_0.25_ alloy, where the B2 matrix phase contains a small amount of the Zr_5_Al_3_ phase particles [34]. The AlNbTiVZr_0.25_ alloy showed a promising combination of properties for potential structural applications, namely low density (5.57 g/cm^3^), high specific strength at room temperature, and 800 °C (244 and 154 kPa m^3^/kg, respectively), and reasonable compression ductility (~10%) at room temperature [34]. The high strength of the alloy at ambient and elevated temperatures was attributed to a strong solid solution strengthening induced by Zr and the B2 ordered matrix phase [34]. In addition, the structure and mechanical properties of the AlNbTiVZr_x_ alloys were found to be quite stable during long-term exposures at 800 °C or 1000 °C [35]. The reason for the sufficiently high phase stability was the presence of Zr, which prevented any phase transformations due to the formation of its own stable phases, like Zr_5_Al_3_ and ZrAlV-type C14 Laves.

In this study, we investigated the oxidation behavior of the AlNbTiVZr_0.25_ high-entropy alloy at potential operating temperatures of 600–900 °C in order to obtain an extended estimation of its performance as a high-temperature material. 

## 2. Materials and Methods

The alloy with a nominal composition of AlNbTiVZr_0.25_ was produced by arc melting of the elements in a low-pressure, high-purity argon atmosphere inside a water-cooled copper cavity. The purities of the alloying elements were no less than 99.9 at.%. The produced ingot of the alloy measured ~6 × 12 × 40 mm^3^. The as-cast ingot was homogenized at 1200 °C for 24 h; hereafter this condition was referred to as the initial one. Prior to the homogenizing, the ingot was encapsulated in a vacuumed (10^−2^ torr) quartz tube filled with titanium chips to prevent any oxidation.

Specimens for oxidation tests with nominal dimensions of 7 × 7 × 2.5 mm^3^ were cut using an electric discharge machine from the ingot with the initial microstructure. Specimen surfaces were subsequently mechanically polished using a MasterMet (Buehler, Lake Bluff, IL, USA) colloidal silica suspension and finally ultrasonically cleaned in isopropanol. Oxidation tests were carried out in a Nabertherm furnace (LT 5/12/P320, Nabertherm, Lilienthal/Bremen, Germany) under static lab air at 600, 700, and 800 °C for 100 h, and 900 °C for 50 h. During the testing process, specimens were placed in alumina crucibles, oxidized at the mentioned temperatures, and removed from the furnace after 0.5, 1, 5, 10, 20, 50, and 100 h of the oxidation test for mass measurements. The mass of specimens before and after oxidation was measured using an HR-200 analytic balance (A&D Company, Ltd., Tokyo, Japan) with an accuracy of 0.1 mg to characterize the oxidation kinetics.

Phase identification of the oxides in the scales was performed by XRD using a RIGAKU Ultima IV diffractometer (Rigaku Corporation, Tokyo, Japan) with Cu-Kα radiation. Additionally, surfaces and cross-sectional morphologies of the specimens were examined by SEM. SEM investigations were carried out using either an FEI Quanta 600 FEG (Thermo Fisher Scientific, Brno, Czech Republic) or a Nova NanoSEM 450 microscope (Thermo Fisher Scientific, Brno, Czech Republic); both instruments were equipped with a back-scattered electron (BSE) and an energy-dispersive X-ray spectroscopy (EDS) detector. The volume fraction of the different phases was measured by a Digimizer Image Analysis Software (MedCalc Software, Ostend, Belgium) using SEM-BSE images.

## 3. Results

### 3.1. Initial Microstructure

**Figure 1:**
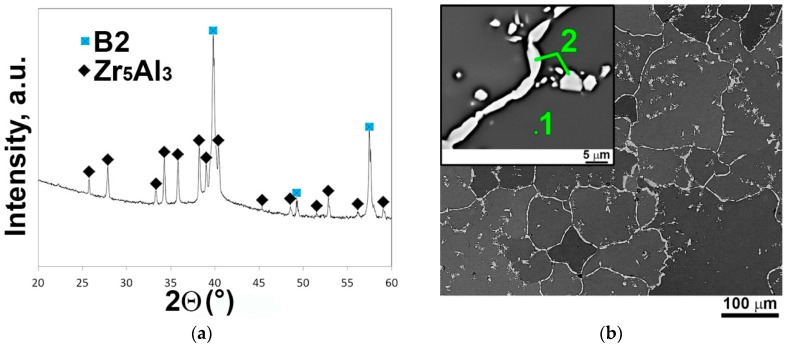
(**a**) X-ray diffraction (XRD) pattern and (**b**) SEM-back-scattered electron (BSE) image of the AlNbTiVZr_0.25_ alloy in the initial state. Chemical compositions of the denoted regions were given in Table 1.

Detailed information on the structure of the AlNbTiVZr_0.25_ alloy has been reported elsewhere [34], thus only a brief description is presented here. Figure 1a shows the XRD pattern of the AlNbTiVZr_0.25_ alloy in the initial (annealed at 1200 °C for 24 h) condition. In the XRD pattern, the Bragg peaks belonging to two phases, namely B2 and Zr_5_Al_3_, were found; the lattice parameters of B2 and Zr_5_Al_3_ phases were a = 0.3203 nm and a = 0.7996 nm, c = 0.5374 nm, respectively. Figure 1b shows the SEM-BSE image of the AlNbTiVZr_0.25_ alloy. The alloy consisted of two structural constituents: (i) B2 grains (labeled with 1 in Figure 1b) with the average size of ~80 μm and the chemical composition close to that of the alloy (Table 1) and (ii) light-grey (Zr, Al)-rich particles (labeled with 2 in Figure 1b; Table 1) identified as the Zr_5_Al_3_ phase located both along the boundaries of the B2 grains as a discontinuous network and as separate particles or clusters of these particles in the B2 grains interior. The estimated volume fraction of the Zr_5_Al_3_ phase was ~5% (Table 1).

### 3.2. Oxidation Behavior

**Figure 2:**
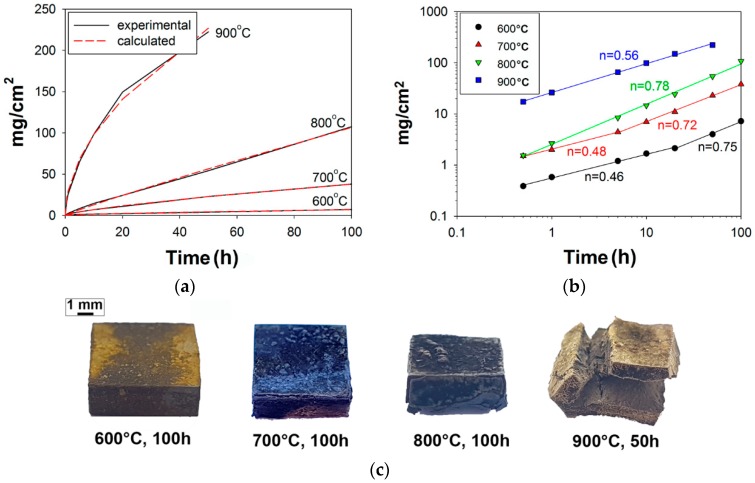
Specific mass gain plots of the AlNbTiVZr_0.25_ alloy at 600, 700, and 800 °C for 100 h, and 900 °C for 50 h in static lab air using (**a**) linear and (**b**) double logarithmic scales; (**c**) images of the samples after testing at 600, 700, and 800 °C for 100 h, and 900 °C for 50 h.

Figure 2a shows the isothermal oxidation kinetics of the AlNbTiVZr_0.25_ alloy at 600, 700, 800, and 900 °C in terms of specific mass gain vs time. Images of the samples after testing at 600, 700, and 800 °C for 100 h, and 900 °C for 50 h are also shown (Figure 2c). Testing at 900 °C was interrupted after 50 h due to the significant disintegration of the sample. After oxidation, the specific mass gains were 7.2, 38.1, and 107.5, and 225.5 mg/cm^2^ at 600, 700, and 800 °C for 100 h, and 900 °C for 50 h, respectively. The mass curves in Figure 2a were fitted to a general oxidation law [36]:

Δm = kt^n^

where Δm is the specific mass gain, k is the oxide growth rate constant, t is time, and n is the time exponent. The best fit for 600 °C and 700 °C was found to be mixed parabolic-linear behavior (n = 0.67 for 600 °C, and n = 0.73 for 700 °C) with k = 0.31 mg/cm^2^ h^0.67^ and k = 1.30 mg/cm^2^ h^0.73^, respectively. The best fit for 800 °C was close to linear (n = 0.92) behavior with k = 1.57 mg/cm^2^ h^0.92^. The rate of mass gain per unit surface area during oxidation at 900 °C followed a near-parabolic dependence with n = 0.52 and k = 29.68 mg/cm^2^ h^0.52^.

The oxidation behavior was also plotted in a double logarithmic scale in Figure 2b. At 600 °C, the alloy demonstrated nearly parabolic kinetics (n = 0.46) in the range of 0–20 h but then the oxidation rate increased significantly (n = 0.75). Similar behavior was found at 700 °C: Nearly parabolic kinetics (n = 0.48) observed from 0 to 5 h changed by mixed parabolic-linear (n = 0.72) one at longer time of testing. At 800 °C, a fairly rapid (n = 0.78) oxidation rate was observed throughout the entire duration of testing. At 900 °C, nearly parabolic kinetics (n = 0.56) was found. The obtained results could suggest the activation of multiple mechanisms during oxidation testing.

### 3.3. Phase Analysis and Morphology of the Surface Layer

**Figure 3:**
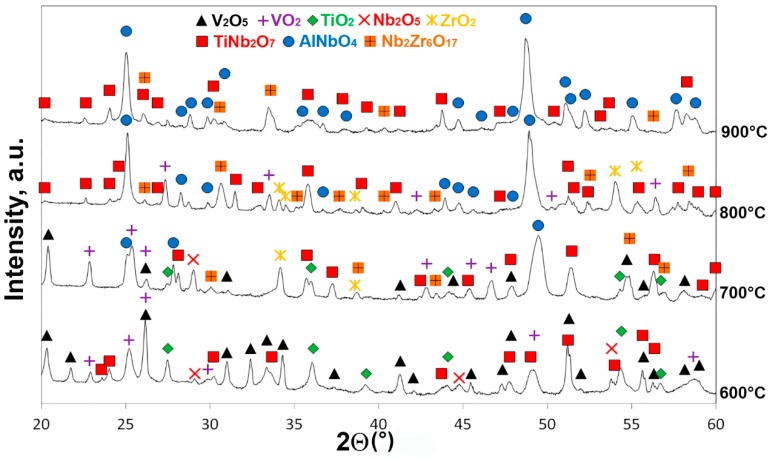
XRD patterns of the surface layers of the AlNbTiVZr_0.25_ alloy oxidized at 600, 700, and 800 °C for 100 h, and 900 °C for 50 h.

Figure 3 presents XRD patterns of the surface layers of the AlNbTiVZr_0.25_ alloy oxidized at 600, 700, and 800 °C for 100 h, and 900 °C for 50 h. In Table 2 the identified oxides and their lattice parameters depending on the temperature are listed. The surface layer of the sample tested at 600 °C was composed of the V_2_O_5_ (space group #59) [37], VO_2_ (space group #130) [38], TiO_2_ (space group #136) [39], Nb_2_O_5_ (space group #15) [40], and TiNb_2_O_7_ (space group #12) [41] oxides; the peaks from the V_2_O_5_ and VO_2_ oxides had the highest intensities. At 700 °C, in addition to the oxides observed at 600 °C, the ZrO_2_ (space group #14) [42], AlNbO_4_ (space group #12) [43], and Nb_2_Zr_6_O_17_ (space group #46) [44] oxides emerged; intensities of the V_2_O_5_ and TiO_2_ peaks were significantly lower in comparison with those at 600 °C. At 800 °C, the TiO_2_, V_2_O_5_, and Nb_2_O_5_ oxides were not detected and the surface layer consisted of the VO_2_ and ZrO_2_ (low-intensity maximums) and TiNb_2_O_7_, AlNbO_4_, and Nb_2_Zr_6_O_17_ (high-intensity maximums) oxides. At 900 °C, the VO_2_ and ZrO_2_ oxides were not detected, and the surface layer was comprised mainly of the complex TiNb_2_O_7_, AlNbO_4_, and Nb_2_Zr_6_O_17_ oxides. Note that the positions of the diffraction peaks from the oxides were very close to that expected from the literature data on their crystal lattice parameters (Table 2).

**Figure 4:**
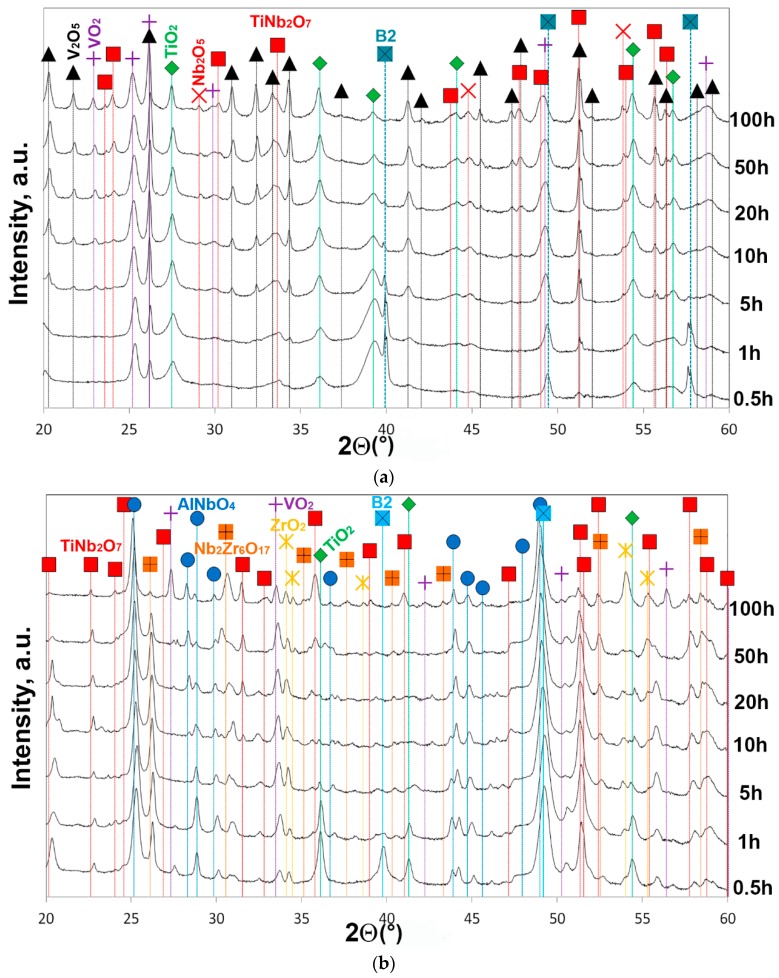
XRD patterns of the surface layers of the AlNbTiVZr_0.25_ alloy oxidized at (**a**) 600 °C and (**b**) 800 °C for 0.5–100 h.

Figure 4 shows the XRD patterns of the surface layers of the samples tested for 0.5–100 h at 600 °C (Figure 4a) and 800 °C (Figure 4b). According to Figure 4a, the initial (0.5 h) stage of oxidation at 600 °C was associated with the formation of the TiO_2_ and VO_2_ oxides; strong diffraction peaks belonging to the B2 phase were also observed. Starting from 5 h exposure, tiny peaks of the V_2_O_5_, TiNb_2_O_7_, and Nb_2_O_5_ oxides were found. The intensity of the oxides peaks, especially V-rich, rose gradually as time progressed. After 20 h, the Braggs peaks of the B2 phase vanished. At 800 °C, the surface layer at the initial (0.5 h) stage of oxidation consisted of the VO_2_, TiO_2_, ZrO_2_, TiNb_2_O_7_, AlNbO_4_, Nb_2_Zr_6_O_17_ oxides and the B2 phase (Figure 4b). Increasing in time over 5 h resulted in the elimination of both the TiO_2_ oxide and the B2 phase from the diffraction patterns. Note that the annealing time had not affected the position of diffraction peaks.

**Figure 5:**
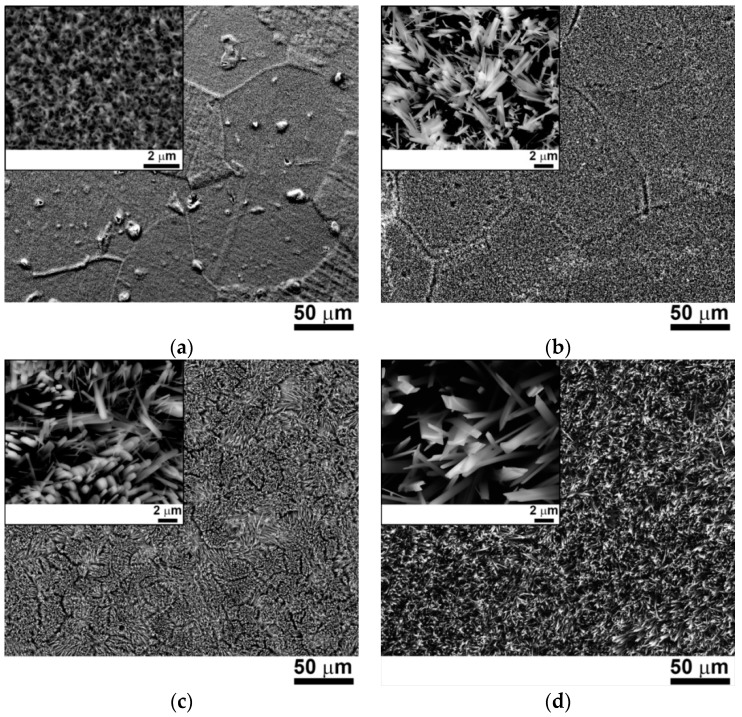
SEM-BSE images of the surface layers of the AlNbTiVZr_0.25_ alloy oxidized for: 0.5 h at (**a**) 600 and (**b**) 800 °C; 100 h at (**c**) 600 and (**d**) 800 °C.

Figure 5 displays SEM-BSE images of the surfaces of the AlNbTiVZr_0.25_ alloy oxidized at 600 and 800 °C for 0.5 and 100 h. Table 3 shows the chemical compositions of the surfaces. The beginning (0.5 h) of the oxidation process occurred differently at 600 and 800 °C. At 600 °C (Figure 5a), the oxidation developed heterogeneously, which is evident from the discrepancy in the topographic appearance of different grains. In addition, very fine oxide nodules were found (magnified insert in Figure 5a). At 800 °C (Figure 5b), the oxidation affected all the grains homogeneously. The oxide whiskers with the average transversal size of ~500 nm were found at the surface (insert in Figure 5b). At 600 °C (0.5 h) the surface layer contained, besides oxygen, almost equal amounts of Al, Nb, Ti, and V, and was depleted of Zr (Table 3). Meanwhile, at 800 °C, the surface layer was predominantly composed of V and O (Table 3). 

After 100 h of oxidation at 600 °C, fine whisker-like oxides with the average transversal size of ~500 nm were found at the surface (Figure 5c). Similar oxide morphology was observed after oxidation at 800 °C (Figure 5d). However, the average transversal size of whiskers was ~1.5 μm. The chemical composition of the surfaces differed from that observed after 0.5 h of oxidation (Table 3). The surface of the sample after 100 h at 600 °C was enriched with V and O, while, nearly equal concentrations of Al, Nb, V, and Ti, and a high O content were found after oxidation at 800 °C (Table 3).

### 3.4. Cross-Sectional Morphologies

**Figure 6:**
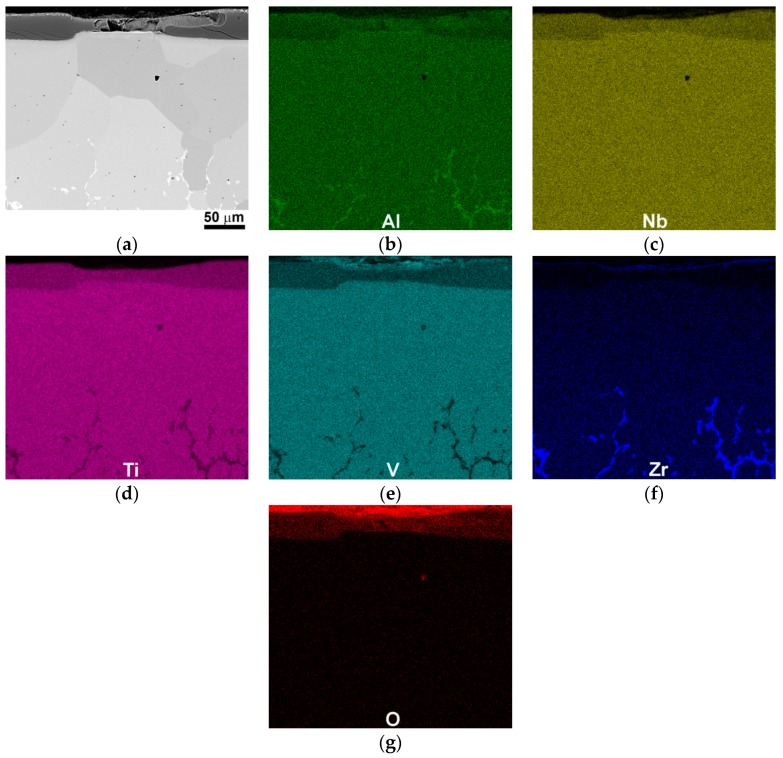
(**a**) SEM-BSE image and (**b**–**g**) corresponded energy-dispersive X-ray spectroscopy (EDS) maps of a cross-section of the AlNbTiVZr_0.25_ alloy after oxidation testing at 600 °C for 100 h.

Figure 6 demonstrates SEM-BSE images and EDS maps of a cross-section of the AlNbTiVZr_0.25_ alloy after oxidation at 600 °C for 100 h. The thickness of the oxide scale varied from ~15 μm to ~30 μm (Figure 6a). Multiple cracks in the oxide scale were found; the cracks seemed to nucleate on the scale-metal interface and propagated towards the surface (Figure 6a). According to the EDS maps (Figure 6b–g), the external part of the oxide scale consisted of a thin (V, Zr)-rich oxide layer (OL) (Figure 6e,f), whereas its inner part was presented by a thick (Ti, Nb, Al)-rich OL (Figure 6b–d). Under the oxide scale, a wide (~100–150 μm) single-phase zone of the substrate with a homogeneous distribution of the constitutive elements was found. Underneath, the material contained particles enriched with Al and Zr (Figure 6b,f), and lean in Ti and V (Figure 6d,e), identified as the initial Zr_5_Al_3_ particles. Many fine pores were found in the material below the scale.

**Figure 7:**
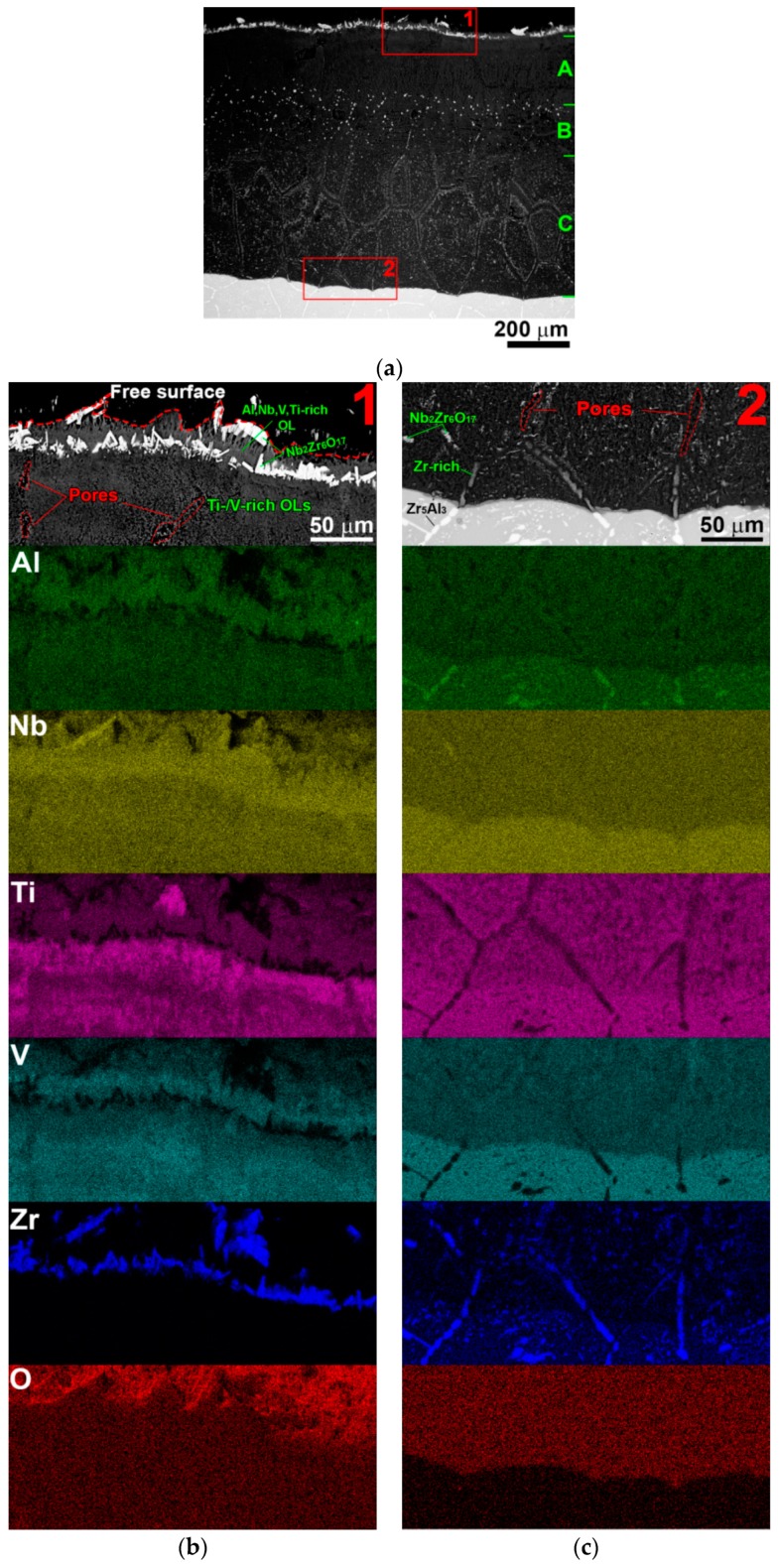
(**a**) SEM-BSE image and (**b**,**c**) combined SEM-BSE-EDS images of (**b**) top part of oxide scale and (**c**) transition zone of a cross-section of the AlNbTiVZr_0.25_ alloy after oxidation testing at 800 °C for 100 h.

At 800 °C, the oxide scale was significantly thicker (~800 μm) and had a complex structure (Figure 7a). The top part of the oxide scale (#1 in Figure 7a,b) consisted of a (Al, Nb, V, Ti)-rich OL in a form of whiskers and a layer of mainly needle-shaped (Zr, Nb)-rich oxide particles. According to XRD data (Figure 3 and Figure 4b), the (Al, Nb, V, Ti)-rich OL could be identified as a mixture of the AlNbO_4_, TiNb_2_O_7_, and VO_2_ oxides, whereas the (Zr, Nb)-rich oxide particles—as the Nb_2_Zr_6_O_17_ oxides. A wide zone with the Ti- and V-rich OLs (“A” in Figure 7a; partly Figure 7b) was observed underneath of the top part; between and inside these OLs, multiple relatively small and separate large pores were found (Figure 7b). The next zone contained numerous relatively small and homogeneously distributed Nb_2_Zr_6_O_17_ oxide particles (“B” in Figure 7a). A thick layer with the regularly spread Nb_2_Zr_6_O_17_ oxide particles was situated directly below and expanding till the substrate (“C” in Figure 7a). The distribution of the particles in this layer was very similar to that of the Zr_5_Al_3_ particles in the substrate (see Figure 1b and Figure 7a).

A close examination of the transition zone (#2 in Figure 7a,c) revealed some notable features. The oxidation front ascended easily along the Zr_5_Al_3_ particles but was slightly impeded by the B2 phase. At the boundary between the oxide scale and the un-oxidized substrate, the former Zr_5_Al_3_ particles started to be depleted with Al which, together with V, segregated to the regions located in the vicinity of these particles. Further, as the oxidation front progressed, Zr-rich particles tended to dissolve with the subsequent formation of the Nb_2_Zr_6_O_17_ oxide particles on the borders of their previous location. In addition, a lot of pores were found at the former sites of the Zr-rich particles (Figure 7c).

## 4. Discussion

### 4.1. Oxidation Kinetics and Mechanisms

The presented results showed that the oxidation behavior of the AlNbTiVZr_0.25_ refractory high-entropy alloy strongly depends on temperature and time. Particularly, at 600–700 °C, the alloy demonstrated a mixed parabolic-linear rate of the mass gain per unit surface area. The same dependences on the double logarithmic scale revealed two distinct stages of oxidation. During the first stage (0–20 h at 600 °C and 0–5 h at 700 °C), oxidation was slow with n = 0.46–0.48. In turn, the second stage (20–100 h at 600 °C and 5–100 h at 700 °C) was characterized by a significantly faster oxidation rate (n = 0.75–0.72). 

Most probably, the acceleration of oxidation at the second stage was caused by an effect similar to breakaway oxidation. The breakaway oxidation occurs if many cracks form continuously and propagate quickly through the oxide scale [45]. The oxide scale cracking/spallation was experimentally observed at 600 °C (Figure 6a) and can probably be associated with the changes in the phase composition of the oxide scale. According to XRD data (Figure 4a), the TiO_2_ and VO_2_, oxides were formed at the beginning of oxidation at 600 °C. Meanwhile, during further (5–10 h) oxidation, the V_2_O_5_, Nb_2_O_5_, and TiNb_2_O_7_ oxides started to appear.

The formation of V-rich oxides and subsequent evaporation is reported to be a common reason for significant deterioration of scale adherence in TiAl-based alloys [46,47,48,49,50]; however, this effect is generally observed at T ≥ 700 °C. It should be noted that the decrease in the quantity and intensity of the V_2_O_5_ oxide diffraction peaks after testing of the AlNbTiVZr_0.25_ alloy at 700 °C can be an indication of its partial evaporation that likely activates the breakaway oxidation at this temperature. 

In turn, T = 600 °C is obviously insufficient for the elimination of the VO_2_ and V_2_O_5_ oxides from the surface. Moreover, our analysis revealed the predominance of the V-rich oxides in the form of whiskers at the gas-oxide interface, whereas cracks were found at the oxide-metal interface (Figure 6). This suggests that the VO_2_ and V_2_O_5_ oxides had an only indirect influence on cracking/spallation at 600 °C, rather than being the main reason. In particular, it can be speculated that the whisker-like morphology of the V-rich oxides accelerates the ingress of oxygen through the oxide scale, and thereby leads to the Nb_2_O_5_ and TiNb_2_O_7_ oxides nucleation in its inner part. The Nb_2_O_5_ oxide can initially form a protective layer but, with the growth of a scale, induces stresses along the oxide-metal interface, resulting in the scale cracking and breakaway oxidation [36,51]. The complex TiNb_2_O_7_ oxide, which most likely appeared due to the reaction of Nb with TiO_2_ and oxygen or as a consequence of the solid-state reaction between Nb_2_O_5_ and TiO_2_ oxides [28,52,53], causes a similar effect [52]. Thus, it can be concluded that the formation of the Nb_2_O_5_ and TiNb_2_O_7_ oxides at 600 °C allows the metal to oxidize according to an undesirable oxygen-alloy interface reaction process [45] due to the continuous cracking/spallation of the oxide scale. 

At 800 °C, the oxidation kinetics obeyed the linear law. This can be due to the formation, from the very beginning of oxidation, of thick, porous oxide scale accompanied by enhanced ingress of gaseous species down to the metal phase [45]. According to XRD analysis, the surface layer consisted, besides the small fraction of the simple ZrO_2_ and VO_2_ oxides, predominantly of the complex oxides such as TiNb_2_O_7_, AlNbO_4_, and Nb_2_Zr_6_O_17_. The similar complex oxides were found after oxidation of the NbTiZrV and AlNbTiZr high-entropy alloys [28,30]. It was reported that the formation of the TiNb_2_O_7_ and Nb_2_Zr_6_O_17_ oxides had not improved oxidation resistance, whereas the AlNbO_4_ oxide formation resulted in sluggish oxidation kinetics. On the contrary, a number of studies demonstrated the detrimental effect of the AlNbO_4_ oxide on the oxidation resistance [53,54,55,56,57].

Apparently, the studied AlNbTiVZr_0.25_ alloy can be considered as Al-free or V-containing analog of the NbTiZrV or AlNbTiZr alloy, respectively. Our results showed that a hypothetical addition of Al to the NbTiZrV does not lead to an enhancement of the oxidation resistance, but only increases the number of the unprotective complex TiNb_2_O_7_ and Nb_2_Zr_6_O_17_ oxides due to the AlNbO_4_ oxide formation. At the same time, a hypothetical doping with V of the AlNbTiZr alloy accelerates oxidation due to the changing of the morphology of the initially protective AlNbO_4_ oxide, and the formation of the complex TiNb_2_O_7_ and Nb_2_Zr_6_O_17_ oxides. Thus, it can be hypothesized that a possible positive effect of Al on the oxidation resistance will be neglected in the presence of V even if Al is added in high amounts. V hinders the formation of protective oxides and facilitates the nucleation of unprotective ones. These reactions, in case of the AlNbTiVZr_0.25_ alloy, are most likely driven by the evaporation of volatile V-rich oxides, particularly, the V_2_O_5_ (Figure 3 and Figure 4b). In addition, the evaporation causes the appearance of multiple pores that serve as places for accelerated ingress of oxygen toward the bare metal and thus stands as the main reason for severe oxidation at 800 °C. It can be assumed that the same processes, but in a more rapid manner, are responsible for the disintegration of the sample tested at 900 °C.

### 4.2. Comparison with the Conventional Alloys and other RHEAs

Table 4 collects data on the specific mass gains (mg/cm^2^) for different alloys, including the examined AlNbTiVZr_0.25_ alloy oxidized at 600–800 °C for 100 h in the air [30,31,50,58,59,60,61,62,63,64,65,66]. The mass gains of the AlNbTiVZr_0.25_ alloy are comparable with some of the V-based alloys, namely V-30Al [59], Ti-based and TiAl-based alloys with a high V content like Ti-35.5V-14.6Cr-0.32Si-0.11C [62], Ti-15V-3Cr-3Sn-3Al [63], and Ti-42Al-8V-(2-4)Mo [50], and the TiZrNbHfTa RHEA [31]. Note that the Hf_0.5_Nb_0.5_Ta_0.5_Ti_1.5_Zr alloy suffered from even more severe oxidation due to pesting [29]. However, the mass gains of many other similar alloys at a given temperature interval are significantly lower. Particularly, the mass gain of the V-free AlNbTiZr RHEA at 600–800 °C is ~5–12 times lower than that of the AlNbTiVZr_0.25_ alloy. This finding confirms that V is largely responsible for the poor oxidation resistance of the AlNbTiVZr_0.25_ alloy. In turn, the mass gains of Inconel 690 or orthorhombic (Ti_2_AlNb) and gamma (TiAl) alloys after oxidation at 800 °C are lower than the experimentally observed values by 3 or 2 orders of magnitude, respectively (Table 4). 

In general, the obtained results demonstrate that despite its promising mechanical properties, the AlNbTiVZr_0.25_ alloy shows rather poor oxidation resistance. This can obviously limit the potential applications of the alloy. To improve the oxidation resistance, modification of the chemical composition, i.e., reduction of the concentration of elements like V or Ti and/or addition or increasing of strong protective scale formers like Al, Cr, or Si is required [23,25,26,27,28,31,33]. The alternative approach is to use protective coatings. Particularly, a recent work [32] reported an effective way to improve the oxidation resistance of the Hf_0.5_Nb_0.5_Ta_0.5_Ti_1.5_Zr RHEA with initially poor oxidation resistance through aluminizing. Nevertheless, the information obtained in the current study can help in the design of RHEAs with a balanced combination of properties including (but not limited to) low density, high strength, sufficient ductility, and good oxidation resistance.

## 5. Conclusions

In this study, the oxidation behavior of the AlNbTiVZr_0.25_ refractory high-entropy alloy at 600–900 °C was investigated. The following conclusions were made:

(1) At 600–700 °C, two-stage oxidation kinetics was found. Nearly parabolic oxidation (n = 0.46–0.48) at the first stage transitioned to breakaway oxidation (n = 0.75–0.72) at the second stage. The breakaway oxidation was induced by spallation of the oxide scale due to the nucleation of voluminous Nb_2_O_5_ and TiNb_2_O_7_ oxides at 600 °C, and probably connected with the partial evaporation of the V_2_O_5_ oxide at 700 °C. At the end of the test (100 h), the surface layers consisted of the V_2_O_5_, VO_2_, TiO_2_, Nb_2_O_5_, TiNb_2_O_7_ oxides at 600 °C, and the V_2_O_5_, VO_2_, TiO_2_, Nb_2_O_5_, TiNb_2_O_7_, ZrO_2_, AlNbO_4_, Nb_2_Zr_6_O_17_ oxides at 700 °C.
(2) At 800 °C, the oxidation kinetics was nearly linear (n = 0.92). The main reason for severe oxidation were the pores, which served as places for accelerated ingress of oxygen toward the bare metal, and appeared as a result of the evaporation of V-rich oxides, and the formation of a mixture of the complex unprotective TiNb_2_O_7_, AlNbO_4_, Nb_2_Zr_6_O_17_ oxides from the very beginning of oxidation. The same oxidation mechanisms were assumed to act at 900 °C that led to the disintegration of the specimen after 50 h.
(3) The oxidation resistance of the alloy can be compared with some V-based/contained alloys. The specific mass gains were estimated to be 7.2, 38.1, and 107.5, and 225.5 mg/cm^2^ at 600, 700, and 800 °C for 100 h, and 900 °C for 50 h, respectively.

## Figures and Tables

**Table 1:** Chemical compositions of the corresponding phases of the AlNbTiVZr_0.25_ alloy in the initial state.

Elements (аt.%)		Al	Nb	Ti	V	Zr	Volume Fraction (%)
Constituents							
No.	Designation						
1	Grains	24.0	23.1	25.2	23.1	4.6	95 ± 2
2	Light-gray particles (Zr_5_Al_3_ phase)	38.2	14.0	11.4	4.4	32.0	5 ± 1
Alloy composition		25.0	22.4	24.0	21.9	6.7	-

**Table 2:** Data on the phase compositions of the surface layers and lattice parameters of the corresponding phases of the AlNbTiVZr_0.25_ alloy oxidized at 600–900 °C.

Oxides	TiO_2_	V_2_O_5_	Nb_2_O_5_	VO_2_	ZrO_2_	TiNb_2_O_7_	AlNbO_4_	Nb_2_Zr_6_O_17_
**Lattice parameter (nm)**	a = 0.4593, c = 0.2959 [39]	a = 0.3565, b = 1.1500, c = 0.4372 [37]	a = 1.2740, b = 0.4883, c = 0.5561 [40]	a = 0.8440, c = 0.7666 [38]	a = 0.5152, b = 0.5208, c = 0.5320 [42]	a = 2.0351, b = 0.3801, c = 1.1882 [41]	a = 1.2157, b = 0.3736, c = 0.6490 [43]	a = 4.0910, b = 0.4930, c = 0.5270 [44]
**Temperature (°C)**								
600	+ ^1^	+	+	+	− ^2^	+	−	−
700	+	+	+	+	+	+	+	+
800	−	−	−	+	+	+	+	+
900 (50 h)	−	−	−	−	−	+	+	+

^1^ oxide is present; ^2^ oxide is absent.

**Table 3:** Chemical compositions of the surface layers of the AlNbTiVZr_0.25_ alloy oxidized at 600 and 800 °C for 0.5 and 100 h.

Elements (аt.%)	Al	Nb	Ti	V	Zr	O
0.5 h						
600 °C	14.1	12.7	12.8	14.7	1.1	44.6
800 °C	15.9	11.0	9.7	23.1	1.2	39.1
100 h						
600 °C	3.6	4.4	4.6	27.9	0.6	58.9
800 °C	13.7	12.8	17.1	12.2	1.0	43.2

**Table 4:** Specific mass gains (in mg/cm^2^) of different alloys oxidized at 600, 700, and 800 °C for 100 h in the air [30,31,50,58,59,60,61,62,63,64,65,66].

Temperature	600 °C	700 °C	800 °C	Reference
AlNbTiVZr_0.25_	7.2	38.1	107.5	This study
AlNbTiZr	1.3 ^1^	5.6 ^1^	8.8 ^1^	[30]
TiZrNbHfTa	-	55	-	[31]
Al_0.3_TiZrNbHfTa	-	14	-	[31]
Al_0.5_TiZrNbHfTa	-	14	-	[31]
Al_0.75_TiZrNbHfTa	-	11	-	[31]
AlTiZrNbHfTa	-	10	-	[31]
Al_9.2_Cr_5.7_Hf_0.5_Mo_1.3_Nb_47.0_Ti_25.9_V_9.6_W_0.8_	-	-	20 ^1^	[58]
V-30Al	-	30	84	[59]
V-30Al-10Cr	-	6	31	[59]
V-30Al-10Ti	-	12	29	[59]
V-5Cr-5Ti	~4.5	-	-	[59]
V-9Si-13B	~3	-	-	[61]
Ti-35.5V-14.6Cr-0.32Si-0.11C	-	-	90	[62]
Ti-6Al-4V	-	-	40	[62]
Grade 2	-	-	26.1 ^2^	[63]
Ti-15V-3Cr-3Sn-3Al	-	-	174.0 ^2^	[63]
Ti-42Al-8V-(2-4)Mo	-	6.7 ^3^	92.3 ^3^	[50]
Ti-48Al-2Cr-2Nb	-	-	0.9	[64]
Ti-22Al-25Nb	-	-	1.1	[65]
Inconel 690	-	-	0.1	[66]

^1^ calculated values; ^2^ oxidized at 815 °C for 96 h; ^3^ 100 cycles.
